# Chronic Kidney Disease and Growth Failure in Children

**DOI:** 10.3390/children11070808

**Published:** 2024-07-01

**Authors:** Tommaso Todisco, Grazia Maria Ubertini, Carla Bizzarri, Sandro Loche, Marco Cappa

**Affiliations:** 1 Research Unit for Innovative Therapies in Endocrinopathies, Bambino Gesù Children’s Hospital, IRCCS, 00165 Rome, Italy; tommaso.todisco@chuv.ch (T.T.);; 2 UOC Endocrinology and Diabetology, Bambino Gesù Children’s Hospital, IRCCS, Piazza S. Onofrio 4, 00165 Rome, Italy

**Keywords:** children chronic kidney disease, growth, growth hormone

## Abstract

Chronic kidney disease (CKD) is a significant challenge for pediatric endocrinologists, as children with CKD may present a variety of endocrine complications. Growth failure is common in CKD, and its severity is correlated with the degree of renal insufficiency. Management strategies include addressing reversible comorbidities, optimizing nutrition, and ensuring metabolic control. Kidney replacement therapy, including transplantation, determines a significant improvement in growth. According to a recent Consensus Statement, children with CKD stage 3—or on dialysis older >6 months—are eligible for treatment with recombinant growth hormone (rGH) in the case of persistent growth failure. Treatment with rGH may be considered for those with height between the 3rd and 10th percentile and persistent growth deceleration. In children who received kidney transplantation but continue to experience growth failure, initiation of GH therapy is recommended one year post-transplantation if spontaneous catch-up growth does not occur and steroid-free immunosuppression is not an option. In children with CKD, due to nephropathic cystinosis and persistent growth failure, GH therapy should be considered at all stages of CKD. Potential adverse effects and benefits must be regularly assessed during therapy. Treatment with GH is safe in children with CKD. However, its general efficacy is still controversial. All possible problems with a negative impact on growth should be timely addressed and resolved, whenever possible with a personalized approach to the patient. GH therapy may be useful in promoting catch-up growth in children with residual growth potential. Future research should focus on refining effective therapeutic strategies and establishing consensus guidelines to optimize growth outcomes in this population.

## 1. Chronic Kidney Disease in Children

Chronic kidney disease (CKD) in the pediatric population is a challenge to physicians. CKD is the compromised ability of the kidney to efficiently remove waste products and excess water and its definition is based upon precise criteria. The prevalence of CKD in children is not negligible with 74.4 cases per million of the age-related population [1,2]. Here, we referred to the KDIGO 2024 Clinical Practice Guideline for the evaluation and management of CKD and for the definition and classification of CKD [3].

A comprehensive understanding of the causes and pathogenesis is essential for the effective management of young patients. The causes of CKD include congenital anomalies, focal segmental glomerulosclerosis, hemolytic uremic syndrome, lupus nephritis, secondary glomerulonephritis, and interstitial nephritis [4]. The clinical presentation of CKD in children encompasses a spectrum of symptoms involving various organ systems. Cardiovascular symptoms may present with hypertension, congestive heart failure, and pericarditis, while gastrointestinal symptoms can manifest as nausea, vomiting, and anorexia. Neurologically, CKD may present with lethargy, confusion, or encephalopathy. Hematologic complications such as anemia and bleeding tendency can occur, and endocrine/metabolic disturbances may manifest with hyperphosphatemia, hypocalcemia, secondary hyperparathyroidism, reduced vitamin D, and growth failure. All these comorbidities may have detrimental effects on growth in children with CKD (Table 1). CKD can also impact sexual and reproductive health by affecting the hypothalamic–pituitary function. Timely recognition of these problems is crucial for appropriate management in pediatric patients with CKD [5].

## 2. Growth Failure in Children with CKD

Growth failure is frequently observed in children with CKD, and the extent of growth impairment is correlated with the degree of renal insufficiency. In the annual report of North American Pediatric Renal Trials and Collaborative Studies 2008 involving 6907 children with CKD, 1/3 presented with a height below −1.88 SDS. In the same study, younger children were more frequently short, with only 17% of children of all ages with CKD with height SDS > 0 [6,7]. Similarly, data from a prospective study on patients from South-East Europe estimated a prevalence of growth failure in 29.3% of the patients with CKD enrolled [7]. Studies on adults have estimated a similar effect of CKD on final height, with reduced height compared to the genetic target in 30 to 50% of patients [7,8,9]. 

Multiple factors contribute to growth failure in children with CKD, including genetic background, nutritional status, mineral and bone disorder, hematological abnormalities, endocrine disturbances, and comorbidities. Anemia and metabolic acidosis, often present in children with CKD, cause poor appetite and consequent malnutrition. However, the factors with the highest impact on growth are the age of onset of CKD and its severity [9]. CKD causes GH resistance and reduced bioactivity of insulin-like growth factor 1 (IGF1). This phenomenon originates from reduced GH receptor expression in the liver and other target organs [10] and abnormalities in GH receptor signaling mediated by the Janus kinase 2–signal transducer and activator of the transcription 5 (STAT5) pathway. IGF1 bioactivity is reduced as measured by sulfate incorporation in an experimental animal model with porcine costal cartilage [11,12] and by the excess of IGF binding proteins (IGFBPs) [13]. In particular, unsaturated IGFBP3, a subclass of the IGFBPs family, has been found to directly inhibit the function of IGF1 on cartilage tissue and on the growth plate chondrocytes in vitro. Furthermore, it has been shown that exogenous GH exerts a positive effect on growth by increasing the levels of IGF1 and its function at the level of the growth plate. All these phenomena are a consequence of the specific uremic state typical of children with CKD.

CKD also directly affects the growth plate metabolism. In CKD rats, the total height of the growth plate has been found reduced, increased, or unchanged when compared to healthy controls. However, when the growth plate was bigger than normal, this resulted from the expansion of the hypertrophic zone that did not correlate with proliferative activity [14]. Additionally, the chondrocytes of CKD rats had a lower final size when compared to controls. This is of great importance since it has been shown that height growth in mammals depends on the volume of chondrocytes of the terminal growth plate [15]. To a certain extent, the reduction in chondrocytes’ final volume may be linked to the relative deficiency of bioactive IGF1 in the growth plate in CKD, as IGF1 is necessary for chondrocytes to achieve their normal final volume [16]. Notably, high-dose rGH therapy in animal models of CKD has been proven to have a beneficial effect on disrupted chondrocytes, namely in terms of the cellular organization of the growth plate and in chondrocyte proliferation [17].

In pediatric patients, CKD determines changes in bone modeling, remodeling, and growth, and these changes manifest early in the progression. CKD–mineral bone disorder (CKD-MBD), a systemic disorder resulting from CKD, is characterized by abnormalities in calcium, phosphorus, parathyroid hormone (PTH), and vitamin D metabolism, resulting in irregularities in bone histology, linear growth, and strength. Secondary hyperparathyroidism also plays a role in growth retardation. However, establishing optimal PTH values for children across all stages of CKD is controversial. In some studies, normal growth velocity has been achieved with PTH levels within the normal range, while in others, higher PTH values were associated with greater growth rates in moderate CKD [18,19]. PTH levels ≥100 pg/mL have been found associated with adynamic bone disease and growth failure in children on maintenance dialysis, indicating that PTH levels should be targeted in advanced CKD [20]. Bone deformities due to altered skeletal remodeling are common findings in uremic children. These may manifest with epiphyseal widening, slipped epiphyses, genu valgum, and femoral and wrist deformities [21]. Avascular necrosis of the femoral head, pathologic fractures, and vertebral crush fractures also significantly contribute to morbidity. The normalization of serum calcium, phosphorus, and PTH levels, with surgical correction as needed after normalization of the biochemical abnormalities, is required in the initial management of skeletal deformities. FGF-23, a phosphaturic hormone, is associated with renal phosphate wasting, low serum 1,25(OH)D3 levels, and osteomalacia in subjects with normal kidney function. FGF-23 levels increase as kidney disease progresses, notably in patients on maintenance dialysis. Yamazaki et al. [22] found that elevated FGF-23 levels were correlated with calcitriol deficiency in adults with CKD stages 2 to 4 and that pre-treatment values may predict resistance of parathyroid glands to vitamin D therapy. These findings indicate that FGF-23 may play a role in serum phosphorus and vitamin D metabolism, and in the pathogenesis of secondary hyperparathyroidism [23,24].

The reproductive system is also affected by CKD. Multiple factors in CKD contribute to the abnormal functioning of the reproductive system. In fact, CKD is associated with a decrease in luteinizing hormone (LH) production and reduced clearance of prolactin, resulting in hyperprolactinemia [25]. Both the elevation of prolactin and the reduction in LH can lead to decreased testosterone production. As CKD progresses to a uremic state, uremia can directly inhibit LH receptors in Leydig cells, thereby impairing testosterone production [26]. In children with CKD, these alterations cause hypogonadism and pubertal delay with detrimental effects on pubertal growth, and possible reduction in adult height [27,28].

Low protein and caloric intake have a negative effect on growth. Balancing caloric intake becomes imperative for optimal growth outcomes in children with CDK. To this purpose, the evaluation of height standard deviation score (SDS), body mass index (BMI), transferrin, and albumin offer important insights into the nutritional status and growth potential of children with CKD. The importance of nutritional status for normal growth in patients with CKD has been confirmed by a Korean study evaluating the risk factors associated with short stature. The study indicated a high prevalence of short stature (23.4%) and underweight (14.1%) in children with CKD and identified underweight as a modifiable risk factor for poor growth [29]. Furthermore, studies in rats have shown that exogenous GH enhances the utilization of caloric intake and increases lean body mass [30].

Poor quality of life (QoL) has been described in children with short stature, especially in cases of chronic kidney disease, achondroplasia, and transfusion-dependent β-thalassemia. This aspect of CKD is often neglected, although it may cause a significant burden to patients and families. The burden for caregivers has been shown to worsen, particularly for parents of children with short stature [31].

## 3. Management of Children with Growth Failure and CKD

Nutritional status is probably the most important factor influencing growth and well-being. Table 2 indicates the strategies for improving nutrition in these children. In the initial assessment of growth, it is essential to estimate the growth potential. Genetic target height (TH) should be calculated. The use of prediction models of adult height is generally not indicated in patients with CKD since these models tend to overestimate adult height.

Growth should be regularly monitored. The use of standardized measurement techniques is recommended, such as supine length using the infantometer before 2 years of age and measuring the Frankfurt vertical plane using a wall-mounted stadiometer. Height and weight should be measured at six-month intervals and plotted on sex- and age-specific reference charts from the same population. The rate of growth rather than stature should be carefully evaluated since CKD may cause growth deceleration in a patient with normal stature. A reduction in growth velocity below the 25th centile represents a significant reduction in growth [27]. All potentially reversible comorbidities with a negative impact on growth should be carefully addressed. This may eventually improve the effect of rGH.

Nutritional interventions, tailored to age, sex, and CKD stage, aimed to align energy and protein intake with dietary reference intake recommendations, should be undertaken as soon as possible, particularly in early childhood and infancy [32]. Metabolic acidosis should be corrected by the administration of oral bicarbonate preparations to maintain HCO3 levels above 23 mmol/L.

Kidney Replacement Therapy (KRT), supported by intensive nutritional care and different dialysis modalities, is generally followed by a significant improvement in growth in end-stage children with CKD. KRT is the optimal therapy in terms of catch-up growth in children with CKD and growth failure, especially if corticosteroid-sparing immunosuppressants are used [33,34]. Clinical trials have shown improved growth after KRT with steroid-sparing regimens like calcineurin inhibitors and the antimetabolite mycophenolate mofetil [35].

## 4. Clinical Practice Recommendations

There is no general consensus on the use of rGH therapy in children with growth failure and CKD. The potential positive effect of rGH therapy should be balanced against its contraindications and possible side effects. In particular, careful calcium/phosphate metabolism monitoring is important, as severe secondary hyperparathyroidism is associated with poor growth and an increased risk of slipped capital femoral epiphysis. The presence of pre-existing papilledema must be ruled out by fundoscopic examination, since rGH therapy may increase the risk of increased intracranial pressure. Active known malignancies and progressive cardiac hypertrophy are absolute contraindications to treatment. Furthermore, rGH is contraindicated in patients with any acute, unstable, or severe conditions. The possible benefit in terms of height gain must be evaluated through the analysis of the residual potential of bone growth and the evaluation of pubertal status. Advanced or delayed bone age and pubertal stage indicate reduced or greater growth potential, respectively [27,36].

The 2019 Consensus Statement [27] recommends the use of rGH in patients with CKD stages 3-5 with growth failure (height velocity <25th centile for age and sex and height below the 3rd centile) persisting for more than 3 months in infants and for more than 6 months in children and older patients. Nevertheless, as in children with CKD and relatively high genetic target, height may slowly progress toward and below the 3rd centile; the Consensus Statements suggest considering rGH therapy in children and infants with CKD stages 3–5, reduction in growth velocity and absolute height between the 3rd and 10th centiles. Considering the lack of data on safety in very young infants, rGH therapy before 6 months is not recommended. For children experiencing persistent growth failure after KT, the Consensus recommends waiting one year after KT to watch for eventual catch-up of growth and consider rGH therapy if growth failure persists and steroid-free immunosuppression therapy is not an option. Patients with nephropathic cystinosis represent a particular condition since they are more prone to develop growth failure despite mild reduction in eGFR. In this case, the Consensus recommends starting rGH therapy as soon as growth failure is manifested at any stage of CKD.

A recent summary of expert opinion has proposed to treat children with CKD independently of eGFR if the underlying cause is distal tubular acidosis, renal Fanconi syndrome, Bartter syndrome, or glomerular disease, in addition to cystinosis. A particular situation is represented by growth failure in pubertal patients with CKD. In these cases, it is recommended to start GH therapy only if bone age is delayed by >1 year [37].

The dose of rGH is greater than that commonly used for patients with GH deficiency and ranges between 0.045 and 0.5 mg/kg/day in daily evening administrations [27]. The dose should be regularly adjusted according to growth and body weight. The response to therapy must be regularly assessed in terms of efficacy and safety. In the presence of persistent headache or vomiting, it is important to stop therapy and evaluate the presence of intracranial hypertension. If rapid growth follows GH therapy, it can increase the risk of slipped capital femoral epiphysis, especially in patients with CKD. In the presence of pain in the groin, inner thigh, or knee, and if a change in gait is observed, discontinuation of therapy and proper evaluation are needed. In case of a positive growth response and absence of adverse effects, treatment should be continued until growth velocity falls below 2 cm/year (when further bone growth is not expected) [27]. Growth failure in CKD is not due to GH deficiency, although relative GH and/or IGF1 insensitivity may play a role. Thus, poor response to exogenous GH is not unusual. There is no clear consensus on the criteria to discontinue treatment in poor responders. According to the Summary of Expert Opinion [37], 52% of experts were in favor of discontinuing rGH therapy if height velocity was below 2 cm/year. On the contrary, 32% of experts were in favor of continuing therapy in the absence of clinical contraindications to rGH. In this scenario, it is advisable to carefully assess the pros and cons of rGH therapy regularly, inform patients and parents, and share any decision to continue or stop treatment.

**Figure 1:**
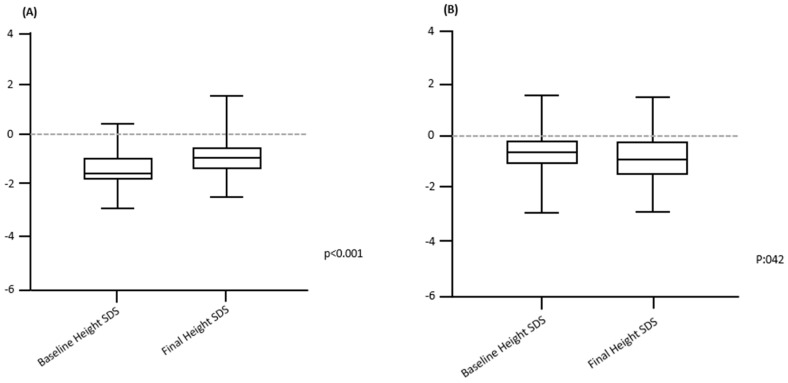
Difference between baseline height SDS and final height SDS (paired t test) in rGH-treated children (**A**) and controls (**B**). Adapted from ref. [39].

A number of studies have shown the beneficial effects of GH therapy in children with stage 3–5 CKD. These data have been recently confirmed by the International Pediatric Peritoneal Dialysis Network [38]. GH treatment was effective in increasing linear growth and weight in infants on chronic peritoneal dialysis despite unsatisfactory caloric intake. This is particularly important since achieving an adequate weight is crucial prior to receiving renal transplantation [39]. The efficacy of the rGH treatment has been demonstrated in a study where the authors found a significant increase in adult height compared to baseline height (Figure 1) [40]. In prepubertal children with severe, end-stage CKD and after renal transplantation, a meta-analysis including 16 randomized control trials (809 patients) showed that after 1 year of therapy, patients on rGH were taller (+0.91 SDS) and had a better growth rate (+3.88 cm/year) than the untreated ones [41]. The expected gain after 2 to 5 years of optimal treatment is estimated to be 7.4 cm in boys and 7 cm in girls [27]. In pubertal patients, data supporting rGH treatment are conflicting. Nevertheless, an increase in adult height has been observed in the majority of them [42,43,44]. Although clinical studies have shown that rGH therapy has a favorable safety profile and increases height gain in children with CKD from stage 3 to dialysis and transplantation, its use is not widespread. This is possibly attributable to the absence of international consensus guidelines. A multidisciplinary approach to the management of children with CKD and growth failure is important to establish the optimal therapeutic option for every single patient.

## 5. Conclusions

Treatment with GH has been approved for the treatment of short stature in children with CKD more than three decades ago. Although current guidelines recommend rGH therapy in all children with CKD and growth deceleration [27], a number of open issues are still pending regarding its use and real efficacy. The effect on adult height is inconsistent and unpredictable [37]. This is probably due to the extreme heterogeneity of the patients. In fact, a number of factors are involved in the pathogenesis of short stature, including the cause of CKD, its duration, steroid therapy, nutrition, hormone resistance, and comorbidities. Some of these factors may be prevalent in some children but not in others. For this reason, the need for personalized therapy in these patients has emerged as a mandatory option. The optimal eGFR level before starting rGH therapy in children older than 1 year with growth failure and CKD is still controversial. In children on maintenance dialysis, PTH levels ≥100 pg/mL are associated with growth failure supporting the recommendation to target PTH levels in advanced CKD [20]. However, it is still debated which level of PTH rGH therapy should not be initiated. Finally, treatment with corticosteroids represents another critical issue in the decision to start or continue rGH therapy and is related to the dose of corticosteroids that would not inhibit the growth-promoting action of rGH. A number of other issues may be present in children who received kidney transplantation and are particularly related to the pre-transplantation disease and to all therapies and complications linked to the transplantation itself.

All possible problems with a negative impact on growth should be timely addressed and resolved whenever possible with a personalized approach to the patient. GH therapy may be useful in promoting catch-up growth in children with residual growth potential.

## Figures and Tables

**Table 1:** The major predictive factors influencing body growth in CKD in children.

Glomerular function baseline < 45 mL/mm/1.73 m^2^
Proteinuria (Up:Uc > 0.2)
Hypertension
Anemia
Hyperparathyroidism
Vitamin D insufficiency and deficiency
Hypocalcemia
Hyperphosphatemia
Metabolic acidosis

**Table 2:** Nutritional assessment and management strategy for children with CKD.

Goal	Maintain Normal Body Mass and Body Composition; Minimize Comorbidities Associated; Slow Progression of Kidney Damage
Achieve good long-term outcomes:	Improve the quality of life; optimize growth
Nutritional assessment:	Body weight for age, height, and height velocity; body mass index normalized protein catabolic rate; protein energy-wasting criteria include evolving body lean mass, biochemistry, diet, and linear growth
Management strategy:	Energy intake same as normal children; nasogastric and gastric tube (PEG), if necessary
Nutrition:	High-carbohydrate content in supplements
Electrolyte balance:	Tubular disorders commonly needing electrolyte supplementation; a low salt diet while hypertension is present; salt supplementation in tubular disorders and high fluid intake in polyuria
